# Exploring the role of immune checkpoint inhibitors in the etiology of myasthenia gravis and Lambert-Eaton myasthenic syndrome: A systematic review

**DOI:** 10.3389/fneur.2022.1004810

**Published:** 2023-01-09

**Authors:** Carly Seligman, Yu-Mei Chang, Jie Luo, Oliver A. Garden

**Affiliations:** ^1^Garden & Luo Immune Regulation Laboratory, Department of Clinical Sciences and Advanced Medicine, School of Veterinary Medicine, University of Pennsylvania, Philadelphia, PA, United States; ^2^Department of Comparative Biomedical Sciences, Royal Veterinary College, University of London, London, United Kingdom; ^3^Dean's Office, School of Veterinary Medicine, Louisiana State University, Baton Rouge, LA, United States

**Keywords:** myasthenia gravis, Lambert-Eaton myasthenic syndrome, autoimmunity, immune checkpoint inhibitors, etiology

## Abstract

**Background:**

While immune checkpoint inhibitors (ICIs) have been revolutionary in the treatment of cancer, their administration has been associated with a variety of immune-related adverse events (irAEs), including myasthenia gravis (MG), and Lambert-Eaton myasthenic syndrome (LEMS).

**Objective:**

To provide a comprehensive synthesis of the evidence supporting an etiological role for ICIs in MG and LEMS in patients with no prior history of autoimmune disease.

**Hypothesis:**

ICIs may trigger MG and LEMS in patients with no prior susceptibility to autoimmune disease.

**Methods:**

Relevant primary research on Medline was interrogated using a series of search algorithms. Search terms were constructed based on the PICOS tool endorsed by the Cochrane Collaboration, which describes population, intervention, comparison, outcomes, and study design. Papers were screened according to inclusion and exclusion criteria. Additional papers were retrieved from the reference lists of screened papers. Each paper included in the qualitative synthesis was assigned an integrated metric of evidence (IME) value, ranging from 0 to 7, based on study design, quality of data, likelihood of a causal link between the immune checkpoint inhibitor(s) and MG/LEMS, confidence of MG/LEMS diagnosis, and the number of patients treated with an ICI prior to MG/LEMS diagnosis.

**Results:**

Ninety-four papers describing at least one patient treated with ICI(s) prior to the onset of MG and/or LEMS were documented. Overall evidence for a causal link between ICI administration and MG/LEMS was low, with a median IME value of 2.88 (range 2.05–6.61).

**Conclusions:**

There is a paucity of evidence in support of an etiological relationship between ICIs and MG/LEMS, due largely to the lack of mechanistic studies and/or prospective clinical trials with relevant study endpoints. The current literature is dominated by case reports and retrospective cohort studies, which inherently yield only low-level evidence, supporting the need for further work in this area. A role of ICIs in the etiology of MG/LEMS remains plausible, arguing for continued vigilance for irAEs in patients treated with these drugs. We argue that there is a need for future mechanistic, high quality, large-scale studies specifically investigating the possible etiological role of ICIs in MG/LEMS.

## Introduction

Immune checkpoint blockade has in recent years become one of the most promising treatments for cancer (1–3). Immune checkpoint inhibitors (ICIs) are monoclonal antibodies that target negative regulators of T cell activation such as programmed death protein-1 (PD-1), programmed death ligand-1 (PD-L1), and cytotoxic T-lymphocyte antigen-4 (CTLA-4) (4). These drugs target the overactive inhibitory T cell pathways in cancer and thus enhance anti-tumor T cell activity (5, 6). While ICIs have yielded promising therapeutic results, their administration has also been associated with numerous immune-related adverse events (irAEs). These irAEs range from minor to severe, with some being life-threatening. One such rare and potentially fatal autoimmune disease is myasthenia gravis (MG) (4, 7–9).

MG is a B-cell mediated autoimmune disease of the neuromuscular junction caused by autoantibodies against acetylcholine receptors (AChR) or, less commonly, muscle-specific kinase (MuSK) or a low-density lipoprotein receptor-related protein (LRP4). This results in dysfunction at the muscle endplate and muscle fatigue and weakness (10). Lambert-Eaton myasthenic syndrome (LEMS) is a junctionopathy clinically resembling MG, in which antibodies directed against presynaptic voltage-gated calcium channels (VGCC) decrease the release of ACh (11, 12). Although T cells are not directly involved in the impairment of neuromuscular transmission in these diseases, CD4^+^ T helper cells (Th cells) have an important pathogenic role by permitting and facilitating the synthesis of high-affinity autoantibodies (13).

Several systematic reviews and meta-analyses investigating the role of ICIs in irAEs have been published (9, 14–21). Past case reports and narrative reviews have also summarized the literature describing an association of ICIs with MG (22–41). However, to the best of our knowledge, a rigorous analysis of the relationship between ICIs and both MG and LEMS has not been undertaken. We have therefore addressed this unmet need by conducting a species-agnostic systematic review of the current literature, in which we interrogate the evidence for a causal relationship between ICIs and MG/LEMS.

## Methods

### Literature review

The literature search was conducted in June 2022. Medline and Web of Science were interrogated for relevant primary research, the former by means of PubMed. Search strings were constructed by means of the “population,” “intervention,” “comparison,” “outcomes,” and “study design” (PICOS) tool endorsed by the Cochrane Collaboration (42). In the case of this systematic review, the “population” comprises patients without a history of autoimmune disease who have received ICIs; the “intervention” is ICI administration; the “comparison” is not applicable as the reviewed papers do not offer contemporaneous data on control patients; the “outcomes” are represented by the presence or absence of MG/LEMS; and the “study design” is incorporated into the IME metric. Papers captured by two primary search algorithms {((myasthenia gravis[MeSH Terms]) OR (myasthenia gravis)) AND (immune checkpoint) NOT (review)} and {((lambert-eaton myasthenic syndrome) OR (lambert eaton myasthenic syndrome)) AND (immune checkpoint) NOT (review)}, denoted as A1 and A2, respectively, were screened according to defined inclusion and exclusion criteria, described in Supplementary material. The search algorithm {(immune checkpoint inhibitors) AND ((adverse events) OR (side effects)) NOT (review)} was used to define ICIs commonly associated with adverse events, after which search algorithms for MG or LEMS and individual ICIs were written. Species-specific search algorithms were written for MG or LEMS and ICIs, including humans, mice, rats, dogs, cats, pigs, horses, cattle, sheep, guinea pigs, baboons, macaques, chimpanzees, chickens, and fish, to ensure that all relevant literature was captured in a manner agonistic of species. Only one paper discussing a non-human species was captured (43). Reference lists of relevant papers and reviews were also screened in order to identify any papers that were not captured by the search algorithms.

### Curation of records

Records were reviewed following the Preferred Reporting Items for Systematic reviews and Meta-Analyses (PRISMA) guidelines (44). Strict observation of the well-defined inclusion and exclusion criteria avoided disputes over which papers to include in the study. All decisions on data accrual were made in a collaborative manner involving CS and OAG; remaining coauthors had access to the entire dataset and were involved in open discussions as data were collected. Multiple opportunities were presented to coauthors during data analysis for discussion of disparate viewpoints, of which none arose.

**Figure 1:**
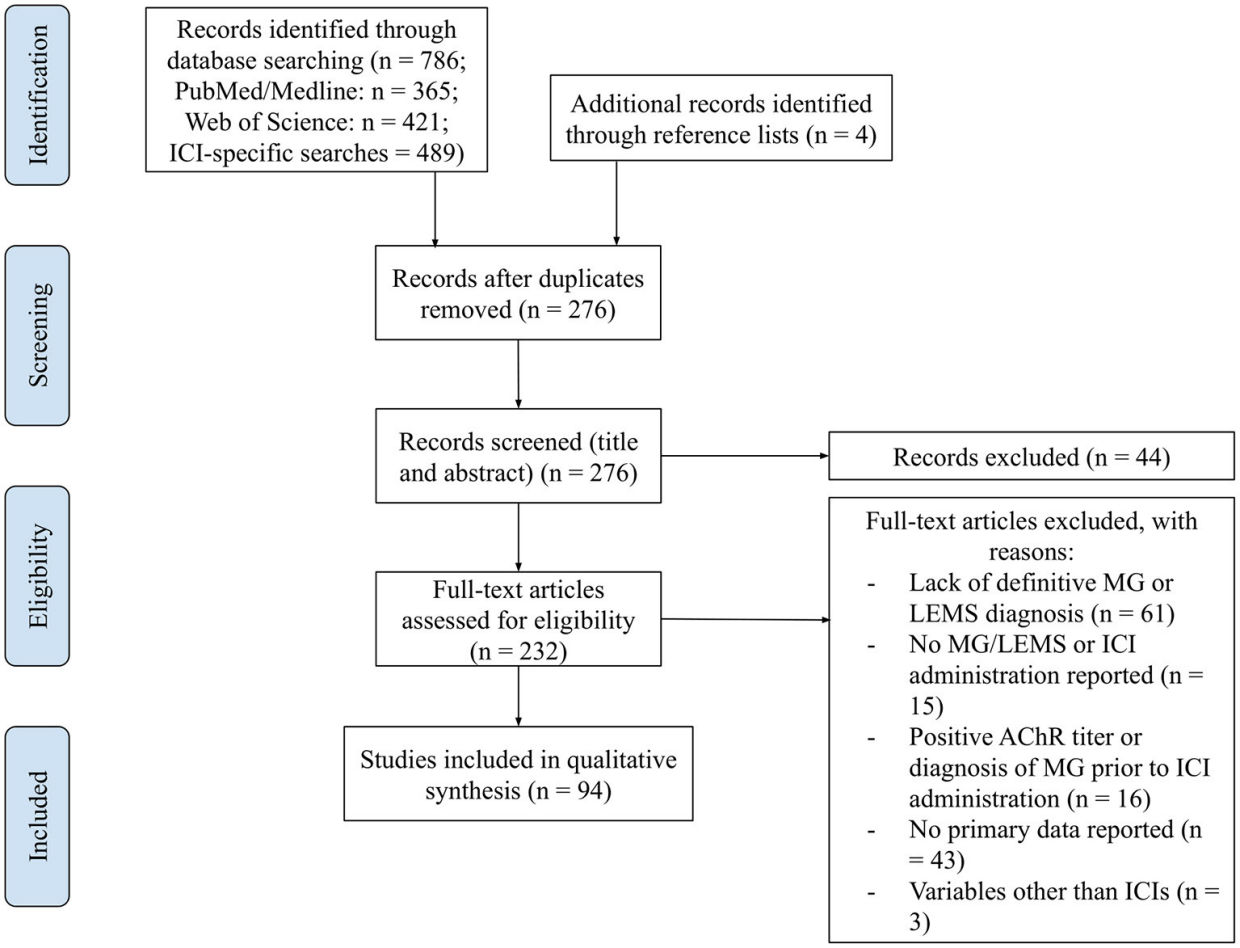
PRISMA flow diagram: curation of records. Papers captured by search algorithms in PubMed (*n* = 786) and additional records identified through reference lists (*n* = 4) were manually curated to remove duplicates (*n* = 276). The titles and abstracts of these papers were screened to assess whether they met the inclusion and exclusion criteria, yielding a total of 232 papers. These full-text articles were then assessed for eligibility, excluding 138 for various reasons. Ninety-four papers were included in the qualitative synthesis.

A total of 786 papers were captured by the search strings A1 and A2, and ICI and species-specific searches (Figure 1). After the removal of duplicates, 276 papers remained. A screening of titles and abstracts removed 44 papers, including irrelevant records and those with pre-existing MG or autoantibodies and lack of primary data. Of the remaining papers, 138 were excluded due to a lack of a definitive diagnosis of MG or LEMS (*n* = 61), no MG/LEMS or ICI administration reported (*n* = 15), a positive AChR titer or diagnosis of MG prior to ICI administration (*n* = 16), a lack of primary data reported (*n* = 43), and/or the administration of therapeutic agents other than ICIs (*n* = 3). The reference lists of records caught by the search strings and reviews were interrogated to identify any papers not captured by the algorithms. Four papers were retrieved, yielding a total of 94 papers for qualitative analysis, including 93 human and one murine study.

### Ethics statement

This systematic review used historical data derived from anonymized patients included in published studies subject to ethical review, precluding the need for *de novo* ethical review or institutional review board approval in this project. No primary samples or prospective data were collected from current human patients.

### Quality assessment

A published assessment instrument called the integrated metric of evidence (IME) designed by Garden et al. was applied to each record (45). This metric assesses study design (D), the quality of the paper (Q), the likelihood of a causal link between the ICI and MG or LEMS (L), the confidence of MG or LEMS diagnosis (I), and the number of patients treated with the ICI(s) (N). The IME value was calculated as a sum of normalized scores for each quantity, weighing each value according to its perceived relevance to evidence rating, using the formula:


$$\begin{array}{l} IME=2D+Q+2L+I+N. \end{array}$$

Each value had a maximum normalized score of 1; therefore, the total maximum IME value was 7. When one value of an individual paper could have multiple scorings, the most parsimonious scores were assigned. Threshold IME values were calculated to categorize associations between ICIs and the onset of MG as being of negligible, low, intermediate, and high evidence. The threshold between negligible and low evidence was taken to be a hypothetical retrospective case report, with a *D* score of 2, *Q* score of 14, *L* and *I* scores of 2, and *N* score of 1 (IME = 2.76). The threshold between low and intermediate evidence was taken to be a hypothetical cross-sectional study with a *D* score of 3, *Q* score of 24, *L* score of 3, *I* score of 2, and *N* score of 2 (IME = 3.96). Lastly, the threshold between intermediate and high evidence was taken to be a hypothetical prospective cohort/case-control study with a *D* score of 5, *Q* score of 28, *L* and *I* scores of 3, and *N* score of 2 (IME = 4.96).

Ten references were selected at random from the pool of papers collected by the search strings, after the removal of duplicates and prior to inclusion/exclusion screening. These papers were independently scored by OAG and CS. The majority of these papers all yielded low level evidence. In order to accommodate papers across the spectrum of evidence, five papers hypothesized to be of higher evidence were specifically selected and independently reviewed by OAG and CS in similar fashion. Agreement between reviewers was evaluated using intraclass correlation.

### Diagnosis of MG and LEMS

**Figure 2:**
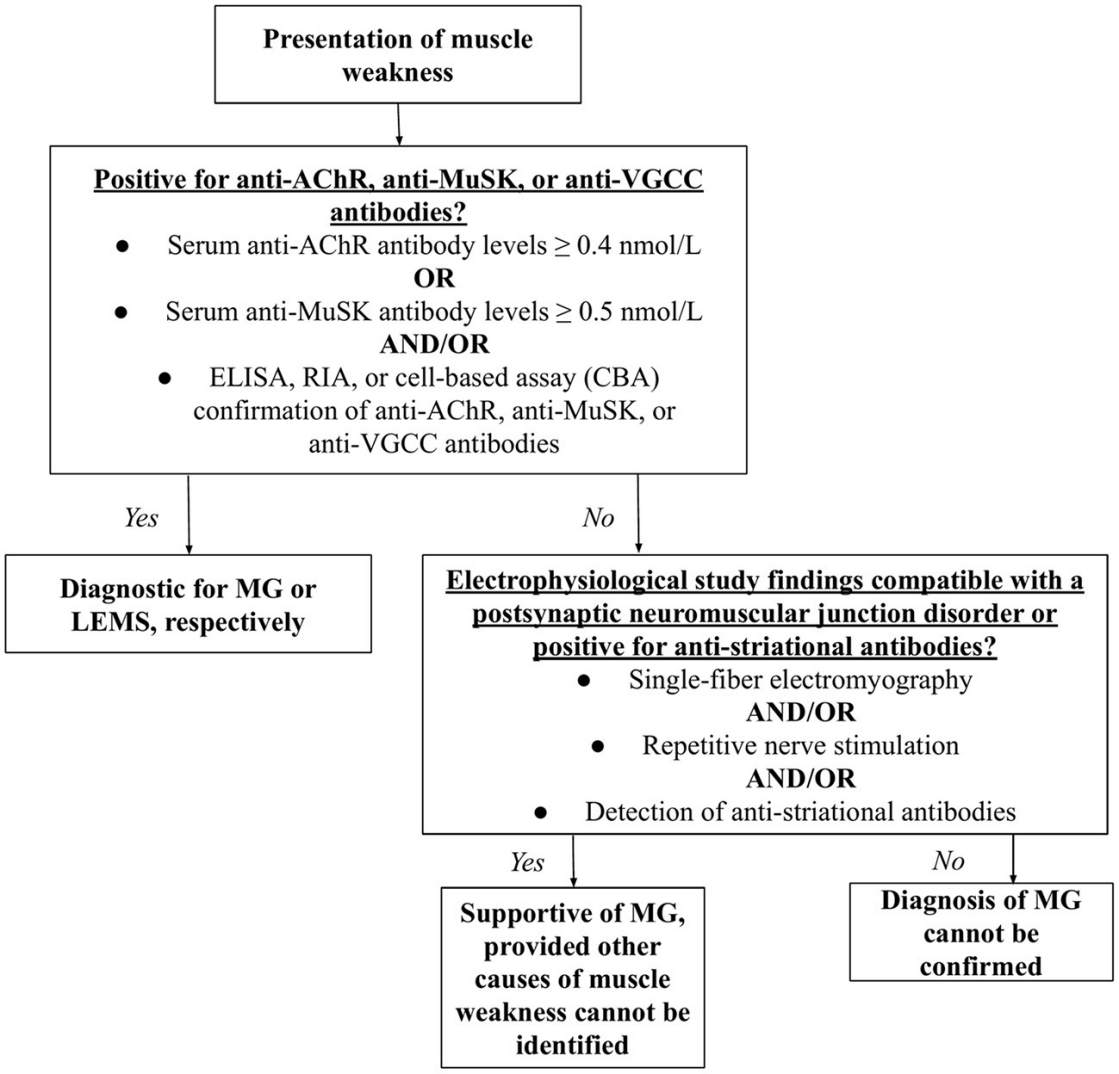
Diagnostic algorithm for MG and LEMS. Having presented with muscle weakness, the patient would be tested for AChR, anti-MuSK, or anti-VGCC antibodies. Detectable levels of such antibodies are diagnostic for MG and LEMS, respectively. If levels of these antibodies are not detectable, single-fiber electromyography, repetitive nerve stimulation, and/or the detection of anti-striational antibodies would support a diagnosis MG, but not LEMS.

The diagnosis of MG or LEMS was made based upon clinical signs and physical examination. Many patients experiencing MG symptoms first complain of muscle weakness and fatigue, after which a serological test is often performed. The detection of anti-AChR or anti-MuSK by cell-based assay (CBA), radioimmunoassay (RIA), or enzyme-linked immunoassay (ELISA) was considered diagnostic for MG, while the presence of anti-VGCC autoantibodies was diagnostic for LEMS (46, 47). Since some MG patients are classified as “triple seronegative,” meaning that neither anti-AChR nor anti-MuSK antibodies are detected, repetitive nerve stimulation (RNS) and single-fiber electromyography (SFEMG) compatible with a postsynaptic neuromuscular junction disorder or the presence of anti-striational antibodies was considered supportive of MG (48–50) (Figure 2).

### Analysis and figures

All analyses were carried out in R (51). Graphs were created using the packages <lattice> and <latticeExtra> (52, 53). Inter-observer correlation coefficient was calculated using the ICC package (54).

### Statistical analysis

A Kruskal-Wallis test was performed to investigate the statistical significance between ICI classes and between individual ICI treatment.

## Results

### Patient data

Out of 220 patients described by papers in this study, information on histological type treated, prior therapy, and ICI administered for 92 was recorded and thus analyzed (Supplementary Table 1). Of these 92 patients, 29 were treated for melanoma, 16 for non-small cell lung cancer (NSCLC), three for small cell lung cancer (SCLC), 16 for renal/urothelial/hepatocellular carcinoma, six for squamous cell carcinoma (SCC), four for thymoma, and 18 for other/unspecified malignancies. Thirty-two patients underwent surgery, 37 chemotherapy, and 18 radiation, either in combination or sole treatment. Six patients received ICIs as first line therapy, and 25 did not report prior therapy.

Paraneoplastic MG/LEMS is a known phenomenon in thymic cancer and SCLC, and was thus considered a confounding variable in these cancer types (11, 55). However, only eight of 92 patients included in our analysis suffered thymic cancer or SCLC in association with MG or LEMS, yielding a negligible impact on overall study conclusions (Supplementary Table 1). In the patients with malignancies other than thymic cancer and SCLC, the probability of paraneoplastic MG/LEMS would have been very low based upon the prevailing literature, arguing against a significant impact on study conclusions. Furthermore, there was no discernible correlation between histological type and MG/LEMS onset in our study cohort.

### Reliability of scoring system

**Figure 3:**
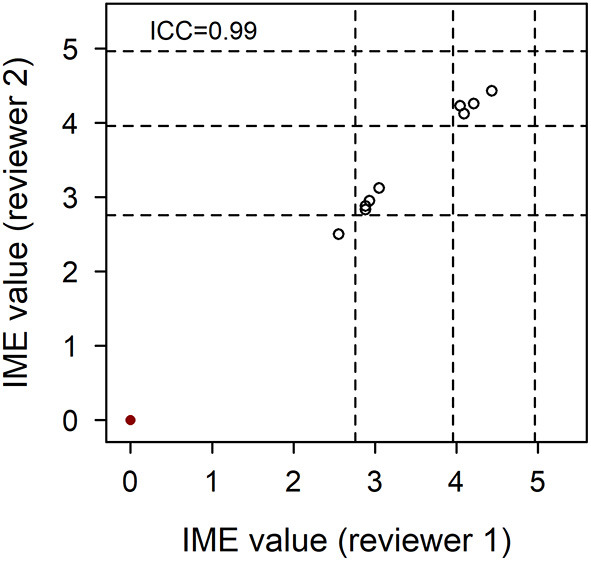
Agreement of integrated metric of evidence (IME) values for 10 randomized and five higher evidence publications. Ten papers from the pool of included records were selected at random and five papers hypothesized to be of higher evidence were selected in targeted fashion. These 15 papers were individually assessed by two reviewers, blinded to each other's scores. The correlation between IME values was 0.99, demonstrating excellent reliability of the scoring system. Horizontal dotted lines indicate the threshold IME values between negligible and low (2.76), low and intermediate (3.96), and intermediate and high (4.96) levels of evidence.

To ensure that the qualitative assessment of papers was independently repeatable between reviewers, 10 papers were selected from the initial pool using a random number generator. Most of these papers yielded negligible to low evidence. Five papers hypothesized to be of higher evidence were therefore targeted to ensure assessment of repeatability across the spectrum of evidence. All 15 papers were scored independently by OAG and CS. The intraclass correlation coefficient for the dataset was 0.99. Furthermore, the IME values of articles assigned by both reviewers fell within 0.2 of each other, confirming excellent reliability of the scoring system (Figure 3).

### Immune checkpoint inhibitors as an etiological trigger

**Figure 4:**
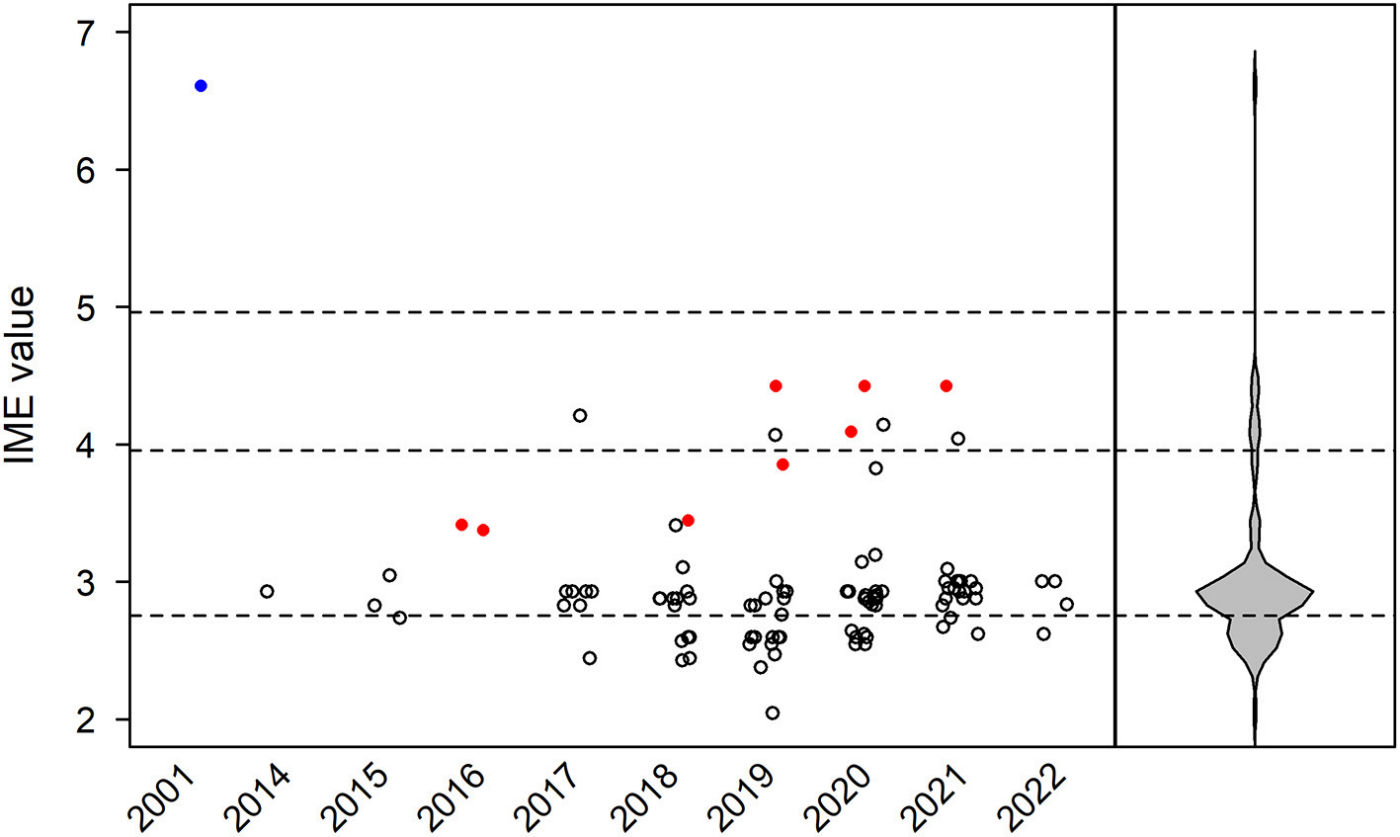
Integrated metric of evidence (IME) values for immune checkpoint inhibitors. Horizontal dotted lines indicate the threshold IME values between negligible and low (2.76), low and intermediate (3.96), and intermediate and high (4.96) levels of evidence. The median IME value was 2.88 (range 2.05–6.61). The sole prospective, blinded, randomized control trial is represented by a blue circle. Papers excluded from formal analysis but considered in our qualitative review are represented by a red circle.

**Figure 5:**
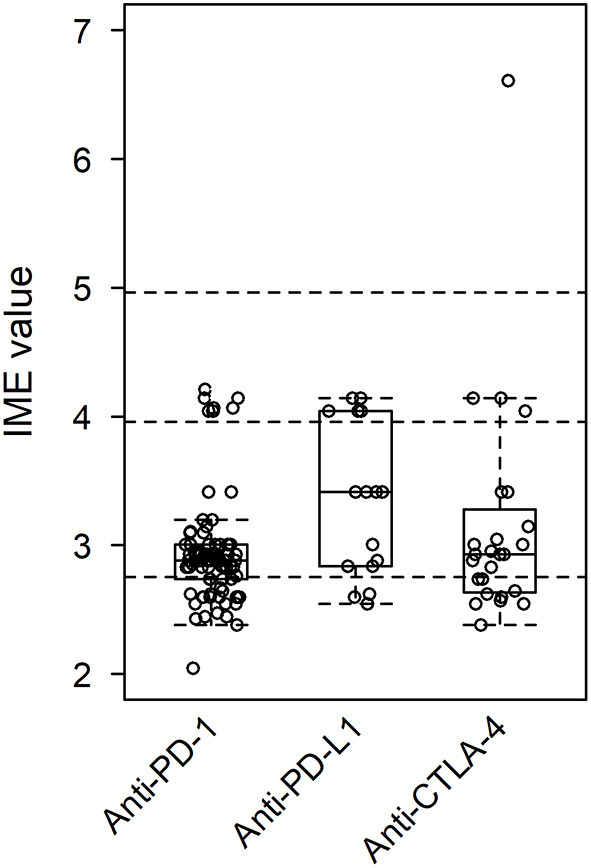
Integrated metric of evidence (IME) values for class of ICI. The median IME values were 2.88, 3.41, and 2.93 for anti-PD-1, anti-PD-L1, and anti-CTLA-4, respectively.

**Figure 6:**
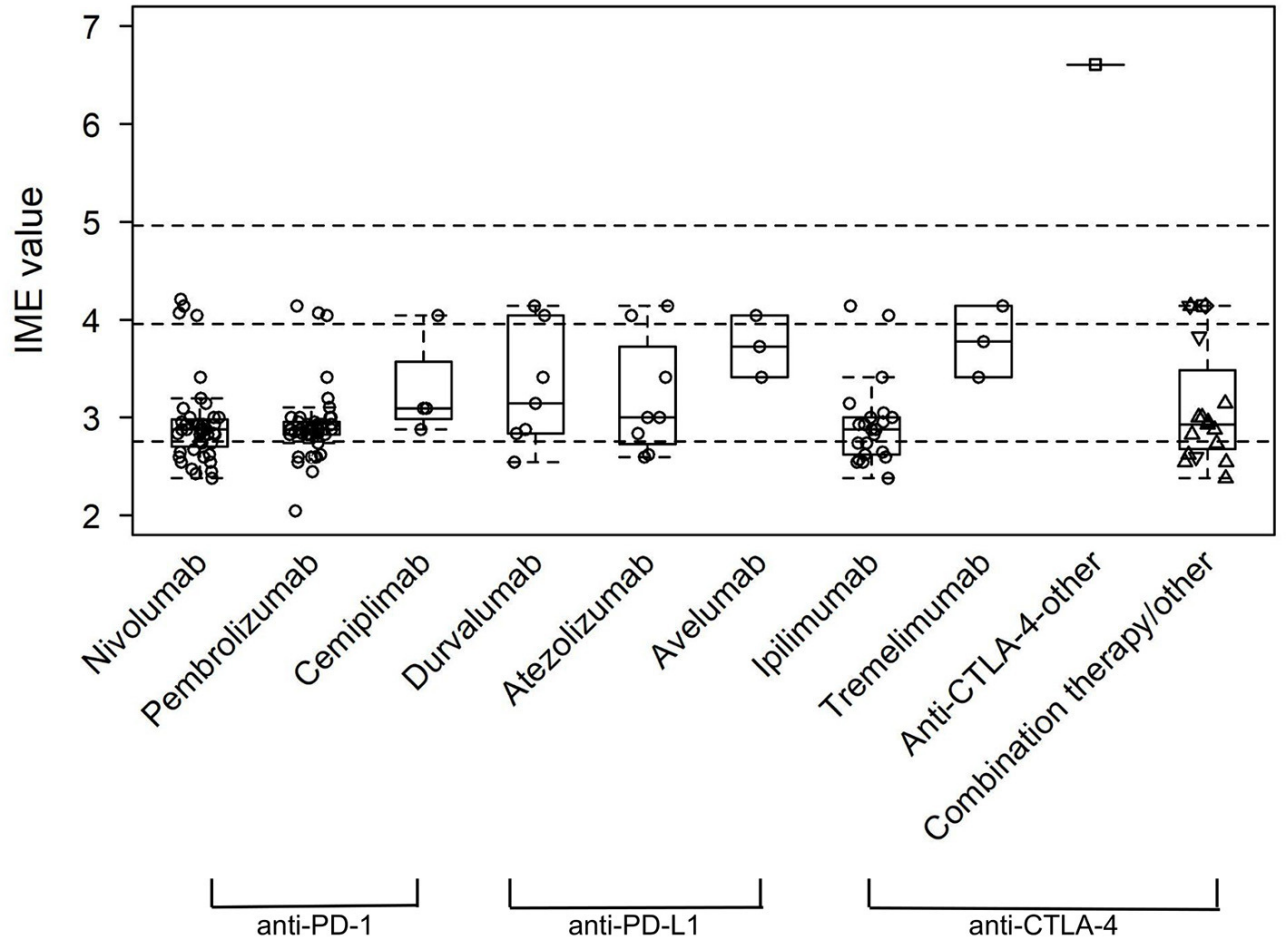
Integrated metric of evidence (IME) values for specific ICIs. The median IME values were 2.88, 2.88, 3.10, 3.00, 3.14, 3.00, 3.73, 2.88, and 3.78 for nivolumab, pembrolizumab, cemiplimab, toripalimab, durvalumab, atezolizumab, avelumab, ipilimumab, and tremelimumab, respectively. For combination therapy/other, other single-agent is represented by a square, nivolumab + ipilimumab by an up-pointing triangle, durvalumab + tremelimumab by a diamond, and other/non-specified combination by a down-pointing triangle.

In total, 94 manuscripts were reviewed (43, 56–147). IME values were calculated for each paper by year of publication (Figure 4). In addition, IME values were computed for three classes of ICI (anti-PD-1, anti-PD-L1, and anti-CTLA-4) and nine individual ICIs (nivolumab, pembrolizumab, cemiplimab, toripalimab, durvalumab, atezolizumab, avelumab, ipilimumab, and tremelimumab) as well as combination therapies (Figures 5, 6). Because most papers directly correlating ICIs with MG or LEMS were retrospective case reports or case series, the median IME values for all categories fell within the low to intermediate range (median = 2.88). Many papers described a link between ICIs and general irAEs instead of focusing on MG or LEMS specifically. In addition, most papers described a correlation of multiple monotherapies and combination therapies with irAEs. Statements are provided for each class of ICI as well as the individual ICIs and combination therapies.

### Anti-PD-1

Of the papers describing a link between the administration of anti-PD-1 drugs and the onset of MG/LEMS, 43 manuscripts mentioned nivolumab, 45 mentioned pembrolizumab, three mentioned cemiplimab, one mentioned toripalimab, and 14 mentioned combination therapies involving an anti-PD-1 drug. The median IME values for nivolumab, pembrolizumab, cemiplimab, and toripalimab association were 2.88, 2.88, 3.10, and 3.00, respectively, while the median IME for papers mentioning combination therapy using at least one anti-PD-1 drug was 2.86. Overall, the IME values ranged from 2.05 to 4.21, with a median of 2.88 (Figures 5, 6). These data suggest low evidence for a causal link between anti-PD-1 administration and MG/LEMS.

### Anti-PD-L1

Six papers mentioned the administration of durvalumab, seven mentioned atezolizumab, and two mentioned avelumab in association with the onset of MG/LEMS. The median IME values for durvalumab, atezolizumab, and avelumab administration were 3.14, 3.00, and 3.73, respectively. One paper mentioned the administration of at least one anti-PD-L1 drug in combination therapy, of which the IME value was 4.14. The IME values ranged from 2.55 to 4.14, with a median of 3.41 (Figures 5, 6). These data suggest low evidence for a causal link between anti-PD-L1 administration and MG/LEMS.

### Anti-CTLA-4

Twenty-one manuscripts mentioned the administration of ipilimumab, while two papers mentioned tremelimumab. The median IME values for papers describing ipilimumab and tremelimumab administration were 2.88 and 3.78, respectively. In addition, 15 papers mentioned an association between combination therapy involving at least one anti-CTLA-4 drug and the onset of MG/LEMS, of which the median IME value was 2.88. Overall, the median IME values for anti-CTLA-4 administration ranged from 2.38 to 6.61, with a median of 2.93. Interestingly, the only manuscript that described induction of experimental autoimmune MG (EAMG) explored possible mechanisms in mice and was thus able to demonstrate a high level of evidence for anti-CTLA-4 antibodies (Figures 5, 6) (43). Overall, this dataset demonstrated low evidence for a causal link between anti-CTLA-4 administration and MG/LEMS.

### Combination therapy

Overall, 20 of the 94 manuscripts mentioned at least one type of combination therapy with or without ICI monotherapy. The most common combination was nivolumab and ipilimumab, mentioned in 13 papers. Fourteen papers mentioned combination therapy with at least one anti-PD-1 drug (median IME value = 2.86), one with at least one anti-PD-L1 drug (IME value = 4.14), and 15 with at least one anti-CTLA-4 drug (median IME value = 2.88; Figure 6). These data suggest low and intermediate evidence for a causal link between anti-PD-1, anti-CTLA-4, and anti-PD-L1 combination therapies and MG/LEMS onset.

### Statistical significance

There was no significant difference between the IME values of ICI classes, of individual ICIs, or of combination therapies (Figures 5, 6). These data suggest no difference in the associations between the administration of distinct ICIs and the onset of MG/LEMS in the evaluated dataset.

## Discussion

ICIs are commonly used to treat cancer. Tumors activate immune checkpoints such as CTLA-4 and PD-1 to inhibit anti-tumor T cell responses (148, 149). ICIs block these checkpoints, thus reversing the attenuation of T cell anti-tumor defense mechanisms (150, 151). While these drugs help in blocking the growth and metastasis of cancer, their inhibition of the immune system can also cause uncontrolled irAEs such as MG and LEMS, prototypical antibody-mediated autoimmune diseases (19). Because ICIs are widely used, understanding their risks to patients, especially those predisposed to autoimmune disease, is important. Owing to the phenomenon of paraneoplastic autoimmune disease, some cancer patients, such as those with thymic cancer or SCLC, could potentially be at increased risk of MG/LEMS with ICI administration (152, 153). For these patients, particular caution may be warranted since ICI administration may trigger the clinical manifestation of autoimmune diseases such as MG and LEMS.

Numerous reviews and meta-analyses have been conducted to elucidate the safety of ICIs. Hottinger outlines the many possible, albeit rare, neurological irAEs known to be associated with ICI administration, highlighting the lack of guidelines available to identify those at risk of developing irAEs (20). In a 2022 meta-analysis, Farooq et al. describe the increased risk of neurological adverse events with the use of ICIs compared to controls, but the ICIs presented less of a risk than chemotherapies (21). As such, while the role of ICIs in irAEs has been investigated, a thorough analysis of their potential role in MG and LEMS has not been undertaken to the best of our knowledge. In order to address this unmet need, we have conducted a systematic review of the literature to investigate the potential role of ICIs in MG/LEMS.

Most the papers included in our qualitative analysis yield a low to intermediate level of evidence in support of a causal link. However, we suggest that this lack of cogent evidence stems from the design of the papers themselves. Many of the studies reviewed were case reports or case series, intrinsically unable to provide convincing evidence. There was a noticeable lack of mechanistic studies that could present higher levels of evidence by virtue of study design. The sole prospective, blinded, randomized control trial that met our inclusion criteria, a paper in which EAMG was induced in mice, yielded an IME value of 6.61, demonstrating high evidence for a causal link (Figure 4). This observation suggests that if more mechanistic studies had been conducted and thus included in our analysis, the median IME value of the dataset would demonstrate higher levels of evidence. While the phenomenon of paraneoplastic autoimmune disease renders it difficult to distinguish between MG/LEMS as paraneoplastic in origin or a consequence of ICI administration in specific individuals, it poses limited relevance in this study since evidence supporting a causal link was lacking; “false positives” were not observed. However, future studies must account for the phenomenon of paraneoplastic MG/LEMS as a potential confounding variable, especially for patients with thymic cancer or SCLC.

Some of the larger studies sourced by our literature search failed to satisfy our inclusion criteria and were therefore excluded from formal analysis but were considered in our qualitative review in order to maximize information derived from the dataset (Figure 4). These studies did not specify the methods of MG/LEMS diagnosis, yet demonstrated some of the attributes necessary for a study to yield strong evidence. Two such papers describe multicenter, open-label phase 1/2 and 1b trials, yielding high scores for quality of study design scores (154, 155). While these studies did not specifically investigate the role of ICIs in MG/LEMS, they demonstrate the rigor of study design necessary for future work specifically focusing on these autoimmune diseases. Six of the papers describe large-scale database studies (156–161), offering the power of large patient populations in drawing conclusions. While the IME values of two of these papers would have fallen in the low to intermediate evidence range when given the most parsimonious I value of 1 (IME = 3.44. 3.85), the other four would have fallen in the intermediate to high range (IME = 4.09, 4.43, 4.43, 4.43). These eight papers thus highlight the potential value of large-scale patient populations, especially when aligned with uniform assessment and diagnostic criteria for MG/LEMS.

In recent years, the administration of ICIs has become a mainstream treatment for cancer (162, 163). Physicians must therefore understand the risks associated with their administration to vulnerable patients. Although irAEs associated with ICIs are rare, the possibility of developing potentially fatal autoimmune diseases such as MG and LEMS warrants future study. While our data suggest no significant differences between ICI classes or individual ICIs in the development of MG/LEMS, future studies would also yield more conclusive inferences on whether some drugs have a greater propensity to induce autoimmune diseases than others. We propose that there is a need for mechanistic and large-scale prospective studies investigating the etiological role of ICIs in MG/LEMS, an important prerequisite in better understanding the risk of these diseases with increasing use of ICIs in clinical medicine.

## Data availability statement

The original contributions presented in the study are included in the article/Supplementary material, further inquiries can be directed to the corresponding author.

## Author contributions

Under the supervision of OG, CS developed the search algorithms, assessed the relevance of all abstracts and full texts, analyzed and interpreted the results, and wrote the first draft of the manuscript. OG analyzed and assessed a subset of articles to ensure concordance of scores. OG and CS developed the scoring system on the basis of a published instrument created by OG and Y-MC. Y-MC helped with statistical analysis and created some of the figures. OG and JL conceptualized the idea for the manuscript and edited the manuscript. All authors contributed to the article and approved the submitted version.
