# Supplementary material for: Exploring the role of immune checkpoint inhibitors in the etiology of myasthenia gravis and Lambert-Eaton myasthenic syndrome: A systematic review
Source: Front Neurol. 2023 Jan 9;13:1004810. doi: 10.3389/fneur.2022.1004810 (PMC9868566; doi:10.3389/fneur.2022.1004810)
Supplement: Supplementary file 1 [file Table_1.pdf]

Author	Journal	Year	Reference #	Patient	ICI Therapy	Prior Therapy	Histological Type	Malignancy
Liao, B., Shroff, S., Kamiya-Matsuoka, C., & Tummala, S.	Neuro Oncol	2014	102	#1	Ipilimumab (anti-CTLA-4)	Surgery	Melanoma	Left uveal metastatic melanoma
Johnson, D. B. et al.	J Clin Oncol	2015	94	#1	Ipilimumab (anti-CTLA-4)	Surgery	Melanoma	Metastatic melanoma
Johnson, D. B. et al.	J Clin Oncol	2015	94	#2	Ipilimumab (anti-CTLA-4)	Surgery	Melanoma	Acrolentiginous melanoma
Loochtan, A. I., Nickolich, M. S., & Hobson-Webb, L. D.	Muscle Nerve	2015	106	#1	Ipilimumab and nivolumab (anti-CTLA-4/anti-PD-1 combination therapy)	Chemotherapy, radiation	SCLC	SCLC
Guidon AC, David W	Muscle & Nerve	2015	84	#1	Nivolumab (anti-PD-1)	N/A	Melanoma	Melanoma
Guidon AC, David W	Muscle & Nerve	2015	84	#2	Ipilimumab and pembrolizumab (anti-CTLA-4/anti-PD-1 combination therapy)	N/A	SCLC	SCLC
Alnahhas, I., Wong, J.	Muscle Nerve	2017	61	#1	Pembrolizumab (anti-PD-1)	Chemotherapy	Melanoma	Metastatic melanoma
Chang, E., Sabichi, A. L., & Sada, Y. H.	J Immunother	2017	66	#1	Nivolumab (anti-PD-1)	Surgery	SCC	Squamous cell carcinoma of the bladder
de Chabot, G., Justeau, G., Pinquié, F., Nadaj-Pakleza, A., Hoppé, E., Hureaux, J., & Urban, T.	Rev Pneumol Clin	2017	71	#1	Nivolumab (anti-PD-1)	Chemotherapy, radiation	NSCLC	Large cell lung carcinoma
Fukasawa, Y. et al.	Case Rep Oncol	2017	79	#1	Nivolumab (anti-PD-1)	N/A	NSCLC	Advanced lung adenocarcinoma
Mehta, J. J., Maloney, E., Srinivasan, S., Seitz, P., & Cannon, M.	Cureus	2017	113	#1	Nivolumab (anti-PD-1)	Surgery, chemotherapy	Renal/urothelial/hepatocellular carcinoma	Metastatic renal cell carcinoma
Tan, R. Y. C., Toh, C. K., & Takano, A.	J Thorac Oncol	2017	140	#1	Nivolumab (anti-PD-1)	Radiotherapy, chemotherapy	NSCLC	NSCLC
Kamada S, Hanazono A, Sanpei Y, Fukunaga H, Inoue T, Suzuki S, et al.	Journal of the Neurological Sciences	2017	95	#1	Nivolumab (anti-PD-1)	N/A	Other/unspecified	Renal cancer
Algaeed, M., Mukharesh, L., Heinzelmann, M., & Kaminski, H.	J. Neurology	2018	59	#1	Pembrolizumab (anti-PD-1)	N/A	Melanoma	Melanoma
Crusz, S. M., Radunovic, A., Shepherd, S., Shah, S., Newey, V., Phillips, M., Lim, L., Powles, T., Szlosarek, P. W., Shamash, J., & Rashid, S.	Eur J Cancer	2018	70	#1	Pembrolizumab (anti-PD-1)	N/A	Melanoma	Unresectable BRAF wild-type in-transit melanoma metastases of his left leg
Fellner, A. et al.	J Neurooncol	2018	76	#7	Pembrolizumab (anti-PD-1)	Surgery	Melanoma	Melanoma (B-RAF wild type), stage-4, lung and brain metastases
Huh, S. Y., et al.	J Clin Neurol	2018	89	#1	Pembrolizumab (anti-PD-1)	Chemotherapy	SCC	Squamous cell carcinoma with metastasis to the pericardium, pleura, and lung
Mancano, M. A., Bulow, J. E. V., & Ro, M.	Hosp Pharm	2018	109	#1	Nivolumab (anti-PD-1)	Surgery, chemotherapy	NSCLC	NSCLC
March, K. L., Samarin, M. J., Sodhi, A., & Owens, R. E.	J Oncol Pharm Pract	2018	110	#1	Pembrolizumab (anti-PD-1)	Radiation therapy	Melanoma	Melanoma of the right scalp with metastases to the liver and brain
Montes, V., Sousa, S., Pita, F., Guerreiro, R., & Carmona, C.	Front Neurol	2018	115	#1	Ipilimumab (anti-CTLA-4)	ICI as first line	Melanoma	Metastatic melanoma
Nakatani, Y., Tanaka, N., Enami, T., Minami, S., Okazaki, T., & Komuta, K.	Case Rep Neurol	2018	117	#1	Nivolumab (anti-PD-1)	Chemotherapy	SCC	Advanced squamous cell lung cancer with multiple bone metastases
Tozuka, T. et al.	Oxf Med Case Reports	2018	143	#1	Pembrolizumab (anti-PD-1)	Surgery	Other/unspecified	Pulmonary pleomorphic carcinoma
Choi YJ, Utset M, Wright R, Soni M	Muscle & Nerve.	2018	67	#1	Ipilimumab (anti-CTLA-4)	Pembrolizumab	Melanoma	Melanoma

Rugiero M, Bettini M, Silveira F, Albacete FS, Christiansen S.	Neuromuscular Disorders.	2018	125	#1	Pembrolizumab (anti-PD-1)	N/A	Melanoma	Melanoma
Jang J, Stream S	Journal of General Internal Medicine.	2018	91	#1	Pembrolizumab (anti-PD-1)	N/A	Renal/urothelial/hepatocellular carcinoma	Renal cell carcinoma
Agrawal, K., Agrawal, N.	Case Rep Neurol Med	2019	57	#1	Ipilimumab and nivolumab (anti-CTLA-4/anti-PD-1 combination therapy)	Chemotherapy, radiation	SCLC	SCLC
Dhenin, A., Samartzi, V., Lejeune, S., & Seront, E.	BMJ Case Rep	2019	72	#1	Pembrolizumab (anti-PD-1)	Surgery	NSCLC	NSCLC
Fazel, M., & Jedlowski, P. M.	Case Reports Immunol	2019	75	#1	Ipilimumab and nivolumab (anti-CTLA-4/anti-PD-1 combination therapy)	Surgery	Melanoma	Metastatic melanoma
Isami, A., Uchiyama, A., Shimaoka, Y., Suzuki, S., Kawachi, I., & Fujita, N.	Rinsho Shinkeigaku	2019	90	#1	Nivolumab (anti-PD-1)	Chemotherapy, radiation	SCC	Squamous cell carcinoma
Kim, J. S., Nam, T. S., Kim, J., Kho, B. G., Park, C. K., Oh, I. J., & Kim, Y. C.	Thorac Cancer	2019	98	#1	Nivolumab (anti-PD-1)	Chemotherapy	NSCLC	NSCLC
Lara, M. S., Afify, A., Ellis, M. P., Phan, C. T., Richman, D. P., & Riess, J. W.	Clin Lung Cancer	2019	101	#1	Pembrolizumab (anti-PD-1)	Chemotherapy	NSCLC	NSCLC
Liu, Q., Ayyappan, S., Broad, A., & Narita, A.	Clin Exp Ophthalmol	2019	104	#1	Pembrolizumab (anti-PD-1)	N/A	Melanoma	Acral lentiginous melanoma
Noda, T., Kageyama, H., Miura, M., Tamura, T., & Ito, H.	Rinsho Shinkeigaku	2019	119	#1	Pembrolizumab (anti-PD-1)	Chemotherapy	NSCLC	Lung adenocarcinoma
Onda, A. et al.	Intern Med	2019	120	#1	Pembrolizumab (anti-PD-1)	ICI as first line	NSCLC	Metastatic lung adenocarcinoma
Rota, E., et al.	eNeurologicalSci	2019	124	#1	Nivolumab (anti-PD-1)	N/A	Other/unspecified	Renal cancer
Rota, E., et al.	eNeurologicalSci	2019	124	#2	Nivolumab (anti-PD-1)	N/A	Other/unspecified	Renal cancer
Sawai, T., Hosokawa, T., Shigekiyo, T., Ogawa, S., Sano, E., & Arawaka, S.	Rinsho Shinkeigaku	2019	127	#1	Nivolumab (anti-PD-1)	Chemotherapy, other	Renal/urothelial/hepatocellular carcinoma	Renal cell carcinoma
Sekiguchi, K. et al.	Muscle Nerve	2019	129	#1	Pembrolizumab (anti-PD-1)	N/A	Other/unspecified	Bladder cancer
Tedbirt, B., De Pontville, M., Branger, P., Picard, C., Baroudjian, B., Lebbé, C., Carpentier, A. F., & Delyon, J.	Eur J Cancer	2019	141	#1	Pembrolizumab (anti-PD-1)	ICI as first line	Melanoma	BRAF wild type stage IV melanoma, unspecified location
Thakolwiboon, S., Karukote, A., & Wilms, H.	Cureus	2019	142	#1	Atezolizumab (anti-PD-L1)	Surgery	Renal/urothelial/hepatocellular carcinoma	Urothelial carcinoma
Werner, J. M., et al.	Front Oncol	2019	148	#1	Ipilimumab and nivolumab (anti-CTLA-4/anti-PD-1 combination therapy)	N/A	Melanoma	Metastatic melanoma
Sutaria R, Patel P, Danve A.	European Journal of Rheumatology	2019	134	#1	Ipilimumab and nivolumab (anti-CTLA-4/anti-PD-1 combination therapy)	Chemotherapy, radiation	Melanoma	Metastatic ocular melanoma
Kankanala VL, Kotecha N.	American Journal of Respiratory and Critical Care Medicine	2019	96	#1	Pembrolizumab (anti-PD-1)	Chemotherapy	Other/unspecified	Urothelial cancer
Sun RQ, Shah V, Tummala S, Chen M	Neurology	2019	133	#1	Nivolumab (anti-PD-1)	N/A	Other/unspecified	Urothelial cancer

Algaeed M, Mukharesh L, Heinzelmann M, Kaminski HJ	Neurology	2019	60	#1	Pembrolizumab (anti-PD-1)	N/A	Melanoma	Melanoma
Diamantopoulos, P. T., et al.	Melanoma Res	2020	73	#1	Ipilimumab (anti-CTLA-4)	ICI as first line	Melanoma	Metastatic malignant melanoma
Diamantopoulos, P. T., et al. Melanoma Res (2020)	Melanoma Res	2020	73	#2	Nivolumab (anti-PD-1)	Surgery	Melanoma	Lentigo maligna melanoma
Fuentes-Antrás, J., et al.	Hematol Oncol Stem Cell Ther.	2020	78	#1	Pembrolizumab (anti-PD-1)	Chemotherapy	NSCLC	Metastatic lung adenocarcinoma
Fukazawa, R., Takezawa, H., Tsuji, Y., Noto, Y., Banba, M., & Fujii, A.	Rinsho Shinkeigaku	2020	80	#1	Pembrolizumab (anti-PD-1)	Chemotherapy, surgery	Other/unspecified	Bladder cancer
Giglio, D., Berntsson, H., Fred, Å., & Ny, L.	Case Rep Oncol	2020	82	#1	Pembrolizumab (anti-PD-1)	Surgery	NSCLC	Adenocarcinoma
Hayakawa, N., Kikuchi, E., Suzuki, S., & Oya, M.	Int Cancer Conf J	2020	85	#1	Pembrolizumab (anti-PD-1)	Chemotherapy, surgery	Renal/urothelial/hepatocellular carcinoma	Metastatic urothelial carcinoma
Ho, A. K., & Cooksley, T.	J Emerg Med	2020	88	#1	Ipilimumab and nivolumab (anti-CTLA-4/anti-PD-1 combination therapy)	Surgery	Renal/urothelial/hepatocellular carcinoma	Metastatic papillary renal cell carcinoma
Jeyakumar, N., Etchegaray, M., Henry, J., Lelenwa, L., Zhao, B., Segura, A., & Buja, L. M.	Case Reports Immunol	2020	93	#1	Cemiplimab (anti-PD-1)	Surgery, radiation	SCC	Squamous cell carcinoma
Lorenzo, C. J., Fitzpatrick, H., Campdesuner, V., George, J., & Lattanzio, N.	Cureus	2020	107	#1	Pembrolizumab (anti-PD-1)	Chemotherapy	Other/unspecified	Malignant mesothelioma
Matas-García, A. et al.	Autoimmun Rev	2020	111	#1	Durvalumab (anti-PD-L1)	N/A	Other/unspecified	Neuroendocrine pancreatic
Mathews, E. P., & Romito, J. W.	Proc (Bayl Univ Med Cent)	2020	112	#1	Ipilimumab and nivolumab (anti-CTLA-4/anti-PD-1 combination therapy)	Surgery	Melanoma	Left ocular melanoma
Miñón-Fernández, B., Losada-Domingo, J. M., Sánchez-Horvath, M. T., & Bárcena-Llona,	J. Rev Neurol	2020	114	#1	Nivolumab (anti-PD-1)	Surgery	Melanoma	Malignant melanoma
Nakanishi, S., Nishida, S., Miyazato, M., Goya, M., & Saito, S.	Urol Case Rep	2020	116	#1	Nivolumab (anti-PD-1)	Chemotherapy, radiation	Renal/urothelial/hepatocellular carcinoma	Renal cell carcinoma
Phua, C. S., Murad, A., Fraser, C., Bray, V., & Cappelen-Smith, C.	BMJ Neurol Open	2020	121	#1	Durvalumab (anti-PD-L1)	Chemotherapy, radiation	NSCLC	Adenocarcinoma
Szuchan, C., Elson, L., Alley, E., Leung, K., Camargo, A. L., Elimimian, E., Nahleh, Z., & Sadler, D.	Eur Heart J Case Rep	2020	137	#1	Pembrolizumab (anti-PD-1)	Chemotherapy, surgery, other, radiation	Other/unspecified	Thymic carcinoma
Takai, M., Kato, D., Iinuma, K., Maekawa, Y. M., Nakane, K., Tsuchiya, T., Yokoi, S., & Koie, T.	Urol Case Rep	2020	139	#1	Pembrolizumab (anti-PD-1)	Chemotherapy	Other/unspecified	Metastatic bladder cancer
Vermeulen, L., Depuydt, C. E., Weckx, P., Bechter, O., Van Damme, P., Thal, D. R., & Claeys, K. G.	Acta Neurol Belg	2020	145	#1	Atezolizumab (anti-PD-L1)	Surgery	Melanoma	Melanoma
Bagley BA, Sigua NL, Snook RJ.	American Journal of Respiratory and Critical Care Medicine	2020	63	#1	Pembrolizumab (anti-PD-1)	Chemotherapy	NSCLC	Adenocarcinoma
Cook C, Moore A, Sharpe M.	Chest	2020	68	#1	Pembrolizumab (anti-PD-1)	Surgery	Other/unspecified	Urothelial cell cancer

Liu SY, Chan J, Brinc D, Gandhi S, Izenberg A, Delgado D, et al.	Jacc: Cardiooncology	2020	105	#1	Pembrolizumab (anti-PD-1)	Surgery	Melanoma	Melanoma
Kovalev D, Muranova A, Patel C.	Annals of Neurology	2020	100	#1	Pembrolizumab (anti-PD-1)	Surgery, chemotherapy, radiation	Thymoma	Thymoma
Canino, F., Pugliese, G., Baldessari, C., Greco, S., Depenni, R., & Dominici, M.	Tumori	2021	64	#1	Cemiplimab (anti-PD-1)	Surgery	SCC	Cutaneous squamous cell carcinoma
Canino, F., Pugliese, G., Baldessari, C., Greco, S., Depenni, R., & Dominici, M.	Tumori	2021	64	#2	Nivolumab (anti-PD-1)	Surgery	Melanoma	Cutaneous melanoma
Gill, A. J., Gandhy, S., & Lancaster, E.	Muscle Nerve	2021	83	#1	Nivolumab (anti-PD-1)	Surgery, chest radiation, chemotherapy	Other/unspecified	Neuroendocrine tumor with small cell features
Ng, A. H., Molinares, D. M., Ngo-Huang, A. T., & Bruera, E.	Ann Palliat Med	2021	118	#1	Nivolumab (anti-PD-1)	N/A	Renal/urothelial/hepatocellular carcinoma	Metastatic clear cell renal cell cancer
Yanase, T., Moritoki, Y., Kondo, H., Ueyama, D., Akita, H., & Yasui, T.	Urol Case Rep	2021	150	#1	Ipilimumab and nivolumab (anti-CTLA-4/anti-PD-1 combination therapy)	Surgery	Renal/urothelial/hepatocellular carcinoma	Renal cell carcinoma
Coustal C, Du Thanh A, Roubille F, Assenat E, Maria ATJ.	European Journal of Cancer	2021	69	#1	Durvalumab (anti-PD-L1)	Chemotherapy	Renal/urothelial/hepatocellular carcinoma	Hepatocellular carcinoma
Heleno CT, Mustafa A, Gotera NA, Tesar A.	Cureus	2021	86	#1	Pembrolizumab (anti-PD-1)	Chemotherapy, radiation	NSCLC	Undifferentiated pancreaticobiliary adenocarcinoma
Serapio C, Saltman A.	Journal of Rheumatology	2021	130	#1	Ipilimumab and nivolumab (anti-CTLA-4/anti-PD-1 combination therapy)	N/A	Renal/urothelial/hepatocellular carcinoma	Urothelial cariconoma
Serapio C, Saltman A.	Journal of Rheumatology	2021	130	#2	Nivolumab (anti-PD-1)	N/A	Melanoma	Melanoma
Luo YB, Tang WT, Zeng QM, Duan WW, Li SY, Yang XS, et al.	Frontiers in Cardiovascular Medicine.	2021	108	#1	Toripalimab (anti-PD-1)	N/A	Thymoma	Thymoma
Figueroa-Perez N, Kashyap R, Bal D, Khan SA, Pattan V.	Cureus.	2021	77	#1	Pembrolizumab (anti-PD-1)	First line	Other/unspecified	Clear cell carcinoma
Verma N, Jaffer M, Pina Y, Peguero E, Mokhtari S.	Cureus Journal of Medical Science	2021	144	#1	Ipilimumab and nivolumab (anti-CTLA-4/anti-PD-1 combination therapy)	N/A	Melanoma	Melanoma
Hernandez AP, Clemente MB, Garcia DE, Calvo RV, Garcia BN, Dominguez JFO, et al.	Cardiovascular Diagnosis and Therapy.	2021	87	#1	Pembrolizumab (anti-PD-1)	Surgery, chemotherapy, radiation	Thymoma	Thymoma
Jespersen MS, Fano S, Stenor C, Moller AK.	European Heart Journal-Case Reports	2021	92	#1	Ipilimumab and nivolumab (anti-CTLA-4/anti-PD-1 combination therapy)	N/A	Renal/urothelial/hepatocellular carcinoma	Renal cell carcihoma
Wakefield C, Shultz C, Patel B, Malla M.	Bmj Case Reports	2021	146	#1	Pembrolizumab (anti-PD-1)	First line	Melanoma	Melanoma
Komatsu M, Hirai M, Kobayashi K, Hashidate H, Fukumoto J, Sato A, et al.	Bmc Gastroenterology	2021	99	#1	Nivolumab (anti-PD-1)	Chemotherapy	Other/unspecified	Gastric cancer
Sanchez-Sancho P, Selva-O'Callaghan A, Trallero-Araguas E, Ros J, Montoro B.	Bmj Case Reports	2021	126	#1	Pembrolizumab (anti-PD-1)	Chemotherapy	Other/unspecified	Liposarcoma
Sugiyama Y, Esa Y, Watanabe A, Kobayashi J, Suzuki S, Takahashi D.	Rinsho Shinkeigaku	2021	132	#1	Ipilimumab and nivolumab (anti-CTLA-4/anti-PD-1 combination therapy)	N/A	Renal/urothelial/hepatocellular carcinoma	Renal cell carcinoma

Portolés Hernández A, Blanco Clemente M, Escribano García D, Velasco Calvo R, Núñez García B, Oteo Domínguez JF, et al.	Cardiovasc Diagn Ther.	2021	122	#1	Pembrolizumab (anti-PD-1)	Surgery, chemotherapy, radiation	Thymoma	Thymoma
Tahir N, Mahboob A, Piao X, Ying G, Shrestha J, Sherchan R, et al.	J Med Cases.	2021	138	#1	Nivolumab (anti-PD-1)	Chemotherapy, radiation	NSCLC	Esophageal adenocarcinoma
Wu D, Condit D, Nascimento JC.	American Journal of Respiratory and Critical Care Medicine.	2021	149	#1	Pembrolizumab (anti-PD-1)	N/A	Other/unspecified	Cervical cancer
Dumortier J, Simon M, Bouhour F.	Clin Res Hepatol Gastroenterol.	2022	74	#1	Atezolizumab (anti-PD-L1)	Chemoembolization, chemotherapy, radiofrequency ablation	Renal/urothelial/hepatocellular carcinoma	Hepatocellular carcinoma
Kee W, Ng KYY, Lee JJX, Tan DSW	Clin Lung Cancer	2022	97	#1	Pembrolizumab (anti-PD-1)	Surgery	NSCLC	NSCLC
Rhee JY, Torun N, Neilan TG, Guidon AC.	Oncologist.	2022	123	#1	Pembrolizumab (anti-PD-1)	Chemotherapy	Renal/urothelial/hepatocellular carcinoma	Renal cell carcinoma
Rhee JY, Torun N, Neilan TG, Guidon AC.	Oncologist.	2022	123	#2	Nivolumab (anti-PD-1)	N/A	Melanoma	Melanoma
Garcia-Garcia J, Diaz-Maroto I, Gonzalez-Villar E, Martinez-Martin A.	Revista De Neurologia.	2022	81	#1	Atezolizumab (anti-PD-L1)	Surgery, chemotherapy	Renal/urothelial/hepatocellular carcinoma	Urothelial carcinoma
