# Supplementary material for: Exploring the role of immune checkpoint inhibitors in the etiology of myasthenia gravis and Lambert-Eaton myasthenic syndrome: A systematic review
Source: Front Neurol. 2023 Jan 9;13:1004810. doi: 10.3389/fneur.2022.1004810 (PMC9868566; doi:10.3389/fneur.2022.1004810)
Supplement: Supplementary file 2 [file Data_Sheet_1.docx]

**Topic:** Impact of immune checkpoint inhibitors (ICIs) as precipitating or predisposing factors in the pathogenesis of myasthenia gravis (MG) and Lambert-Eaton myasthenic syndrome (LEMS) in all species.

**Question:** How do ICIs impact the pathogenesis and prognosis of MG and LEMS in all species? Do ICIs trigger the onset and propagation MG and LEMS?

**Hypothesis:** We hypothesize that ICIs trigger the onset and propagation of MG and LEMS in all species.

**Inclusion criteria:**

1. The study describes at least one ICI administered preceding diagnosis of MG and LEMS.
2. The study reports primary data. Reports of individual cases are suitable if they otherwise fulfill the remaining inclusion criteria.
3. The study is published in complete form in a peer-reviewed journal between 1978, following the establishment of an autoimmune response against muscle AChR in MG, and June of 2022. If written in a language other than English, it will be translated to assess suitability.
4. MG is diagnosed based on compatible clinical features (i.e. fluctuating fatigability and weakness affecting ocular, bulbar and proximal limb skeletal muscle groups) together with ne or more of the following criteria:
   1. Serum anti-AChR antibody levels ≥ 0.4 nmol/L
   2. Serum anti-MuSK antibody levels ≥ 0.5 nmol/L
   3. ELISA, RIA, or cell-based assay (CBA) confirmation of anti-AChR or anti-MuSK antibodies
   4. Electrophysiological study findings compatible with a postsynaptic neuromuscular junction disorder (repetitive stimulation, single-fiber electromyography, or both) or the presence of anti-striational antibodies
5. LEMS is diagnosed based on compatible clinical features together with anti-VGCC antibodies.

**Exclusion criteria:**

1. Papers that described ICI administration succeeding detection of anti-AChR/anti-MuSK/anti-VGCC antibodies or MG/LEMS diagnosis were excluded.
2. Papers that were published prior to 1978 were excluded.

**Refined searches in PubMed:**

MG:

- ((myasthenia gravis[MeSH Terms]) OR (myasthenia gravis)) AND (immune checkpoint) NOT (review)
- ((myasthenia gravis[MeSH Terms]) OR (myasthenia gravis)) AND (immune checkpoint) AND (review)
- (immune checkpoint inhibitors) AND ((adverse events) OR (side effects)) NOT (review)
- ((myasthenia gravis[MeSH Terms]) OR (myasthenia gravis)) AND (anti-PD-1) NOT (review)
- ((myasthenia gravis[MeSH Terms]) OR (myasthenia gravis)) AND (anti-PD-L1) NOT (review)
- ((myasthenia gravis[MeSH Terms]) OR (myasthenia gravis)) AND (anti-CTLA-4) NOT (review)
- ((myasthenia gravis[MeSH Terms]) OR (myasthenia gravis)) AND ((ipilimumab) OR (BMS-734016) OR (MDX-010) OR (MDX-101)) NOT (review)
- ((myasthenia gravis[MeSH Terms]) OR (myasthenia gravis)) AND ((nivolumab) OR (ONO-4538) OR (BMS-936558) OR (MDX1106)) NOT (review)
- ((myasthenia gravis[MeSH Terms]) OR (myasthenia gravis)) AND ((pembrolizumab) OR (MK-3475) OR (lambrolizumab)) NOT (review)
- ((myasthenia gravis[MeSH Terms]) OR (myasthenia gravis)) AND ((atezolizumab) OR (MPDL3280A) OR (RG7446)) NOT (review)
- ((myasthenia gravis[MeSH Terms]) OR (myasthenia gravis)) AND ((durvalumab) OR (MEDI4736) OR (MEDI-4736)) NOT (review)
- ((myasthenia gravis[MeSH Terms]) OR (myasthenia gravis)) AND ((avelumab) OR (MSB0010718C)) NOT (review)
- ((myasthenia gravis[MeSH Terms]) OR (myasthenia gravis)) AND ((tremelimumab) OR (ticilimumab)) NOT (review)
- ((myasthenia gravis[MeSH Terms]) OR (myasthenia gravis)) AND (pidilizumab) NOT (review)
- ((myasthenia gravis[MeSH Terms]) OR (myasthenia gravis)) AND ((cemiplimab) OR (REGN-2810) OR (REGN2810) OR (cemiplimab-rwlc)) NOT (review)
- ((myasthenia gravis[MeSH Terms]) or (myasthenia gravis)) AND ((anti-PD-1) OR (anti-PD-L1) OR (anti-CTLA-4)) AND (anti-LRP4) NOT (review)

LEMS:

- ((lambert-eaton myasthenic syndrome) OR (lambert eaton myasthenic syndrome)) AND (immune checkpoint) NOT (review)
- ((lambert-eaton myasthenic syndrome) OR (lambert eaton myasthenic syndrome)) AND (anti-PD-1) NOT (review)
- ((lambert-eaton myasthenic syndrome) OR (lambert eaton myasthenic syndrome)) AND (anti-PD-L1) NOT (review)
- ((lambert-eaton myasthenic syndrome) OR (lambert eaton myasthenic syndrome)) AND (anti-CTLA-4) NOT (review)
- ((lambert-eaton myasthenic syndrome) OR (lambert eaton myasthenic syndrome)) AND ((ipilimumab) OR (BMS-734016) OR (MDX-010) OR (MDX-101)) NOT (review)
- ((lambert-eaton myasthenic syndrome) OR (lambert eaton myasthenic syndrome)) AND ((nivolumab) OR (ONO-4538) OR (BMS-936558) OR (MDX1106)) NOT (review)
- ((lambert-eaton myasthenic syndrome) OR (lambert eaton myasthenic syndrome)) AND ((pembrolizumab) OR (MK-3475) OR (lambrolizumab)) NOT (review)
- ((lambert-eaton myasthenic syndrome) OR (lambert eaton myasthenic syndrome)) AND ((atezolizumab) OR (MPDL3280A) OR (RG7446)) NOT (review)
- ((lambert-eaton myasthenic syndrome) OR (lambert eaton myasthenic syndrome)) AND ((durvalumab) OR (MEDI4736) OR (MEDI-4736)) NOT (review)
- ((lambert-eaton myasthenic syndrome) OR (lambert eaton myasthenic syndrome)) AND ((avelumab) OR (MSB0010718C)) NOT (review)
- ((lambert-eaton myasthenic syndrome) OR (lambert eaton myasthenic syndrome)) AND ((tremelimumab) OR (ticilimumab)) NOT (review)
- ((lambert-eaton myasthenic syndrome) OR (lambert eaton myasthenic syndrome)) AND (pidilizumab) NOT (review)
- ((lambert-eaton myasthenic syndrome) OR (lambert eaton myasthenic syndrome)) AND ((cemiplimab) OR (REGN-2810) OR (REGN2810) OR (cemiplimab-rwlc)) NOT (review)

Murine/mice/mouse/rat/rodent:

- ((myasthenia gravis[MeSH Terms]) OR (myasthenia gravis)) AND (immune checkpoint) AND ((murine) OR (mice) OR (mouse) OR (rat) OR (rodent)) NOT (review)
- ((myasthenia gravis[MeSH Terms]) OR (myasthenia gravis)) AND ((anti-PD-1) OR (anti-PD-L1) OR (anti-CTLA-4)) AND ((murine) OR (mice) OR (mouse) OR (rat) OR (rodent)) NOT (review)
- ((myasthenia gravis[MeSH Terms]) OR (myasthenia gravis)) AND (immune checkpoint) AND (EAMG) NOT (review)
- ((myasthenia gravis[MeSH Terms]) OR (myasthenia gravis)) AND ((ipilimumab) OR (BMS-734016) OR (MDX-010) OR (MDX-101)) AND ((murine) OR (mice) OR (mouse) OR (rat) OR (rodent)) NOT (review)
- ((myasthenia gravis[MeSH Terms]) OR (myasthenia gravis)) AND ((nivolumab) OR (ONO-4538) OR (BMS-936558) OR (MDX1106)) AND ((murine) OR (mice) OR (mouse) OR (rat) OR (rodent)) NOT (review)
- ((myasthenia gravis[MeSH Terms]) OR (myasthenia gravis)) AND ((pembrolizumab) OR (MK-3475) OR (lambrolizumab)) AND ((murine) OR (mice) OR (mouse) OR (rat) OR (rodent)) NOT (review)
- ((myasthenia gravis[MeSH Terms]) OR (myasthenia gravis)) AND ((atezolizumab) OR (MPDL3280A) OR (RG7446)) AND ((murine) OR (mice) OR (mouse) OR (rat) OR (rodent)) NOT (review)
- ((myasthenia gravis[MeSH Terms]) OR (myasthenia gravis)) AND ((durvalumab) OR (MEDI4736) OR (MEDI-4736)) AND ((murine) OR (mice) OR (mouse) OR (rat) OR (rodent)) NOT (review)
- ((myasthenia gravis[MeSH Terms]) OR (myasthenia gravis)) AND ((avelumab) OR (MSB0010718C)) AND ((murine) OR (mice) OR (mouse) OR (rat) OR (rodent)) NOT (review)
- ((myasthenia gravis[MeSH Terms]) OR (myasthenia gravis)) AND ((tremelimumab) OR (ticilimumab)) AND ((murine) OR (mice) OR (mouse) OR (rat) OR (rodent)) NOT (review)
- ((myasthenia gravis[MeSH Terms]) OR (myasthenia gravis)) AND (pidilizumab) AND ((murine) OR (mice) OR (mouse) OR (rat) OR (rodent)) NOT (review)
- ((myasthenia gravis[MeSH Terms]) OR (myasthenia gravis)) AND ((cemiplimab) OR (REGN-2810) OR (REGN2810) OR (cemiplimab-rwlc)) AND ((murine) OR (mice) OR (mouse) OR (rat) OR (rodent)) NOT (review)

Dog/cat:

- ((myasthenia gravis[MeSH Terms]) OR (myasthenia gravis)) AND (immune checkpoint) AND ((dog) OR (canine) OR (cat) OR (feline)) NOT (review)
- ((myasthenia gravis[MeSH Terms]) OR (myasthenia gravis)) AND ((anti-PD-1) OR (anti-PD-L1) OR (anti-CTLA-4)) AND ((dog) OR (canine) OR (cat) OR (feline)) NOT (review)
- ((myasthenia gravis[MeSH Terms]) OR (myasthenia gravis)) AND ((ipilimumab) OR (BMS-734016) OR (MDX-010) OR (MDX-101)) AND ((dog) OR (canine) OR (cat) OR (feline)) NOT (review)
- ((myasthenia gravis[MeSH Terms]) OR (myasthenia gravis)) AND ((nivolumab) OR (ONO-4538) OR (BMS-936558) OR (MDX1106)) AND ((dog) OR (canine) OR (cat) OR (feline)) NOT (review)
- ((myasthenia gravis[MeSH Terms]) OR (myasthenia gravis)) AND ((pembrolizumab) OR (MK-3475) OR (lambrolizumab)) AND ((dog) OR (canine) OR (cat) OR (feline)) NOT (review)
- ((myasthenia gravis[MeSH Terms]) OR (myasthenia gravis)) AND ((atezolizumab) OR (MPDL3280A) OR (RG7446)) AND ((dog) OR (canine) OR (cat) OR (feline)) NOT (review)
- ((myasthenia gravis[MeSH Terms]) OR (myasthenia gravis)) AND ((durvalumab) OR (MEDI4736) OR (MEDI-4736)) AND ((dog) OR (canine) OR (cat) OR (feline)) NOT (review)
- ((myasthenia gravis[MeSH Terms]) OR (myasthenia gravis)) AND ((avelumab) OR (MSB0010718C)) AND ((dog) OR (canine) OR (cat) OR (feline)) NOT (review)
- ((myasthenia gravis[MeSH Terms]) OR (myasthenia gravis)) AND ((tremelimumab) OR (ticilimumab)) AND ((dog) OR (canine) OR (cat) OR (feline)) NOT (review)
- ((myasthenia gravis[MeSH Terms]) OR (myasthenia gravis)) AND (pidilizumab) AND ((dog) OR (canine) OR (cat) OR (feline)) NOT (review)
- ((myasthenia gravis[MeSH Terms]) OR (myasthenia gravis)) AND ((cemiplimab) OR (REGN-2810) OR (REGN2810) OR (cemiplimab-rwlc)) AND ((dog) OR (canine) OR (cat) OR (feline)) NOT (review)

Pig/horse/cattle/sheep:

- ((myasthenia gravis[MeSH Terms]) OR (myasthenia gravis)) AND (immune checkpoint) AND ((pig) OR (suine) OR (horse) OR (equine) OR (cow) OR (cattle) OR (bovine) OR (sheep) OR (ovine)) NOT (review)
- ((myasthenia gravis[MeSH Terms]) OR (myasthenia gravis)) AND ((anti-PD-1) OR (anti-PD-L1) OR (anti-CTLA-4)) AND ((pig) OR (suine) OR (horse) OR (equine) OR (cow) OR (cattle) OR (bovine) OR (sheep) OR (ovine)) NOT (review)
- ((myasthenia gravis[MeSH Terms]) OR (myasthenia gravis)) AND ((ipilimumab) OR (BMS-734016) OR (MDX-010) OR (MDX-101)) AND ((pig) OR (suine) OR (horse) OR (equine) OR (cow) OR (cattle) OR (bovine) OR (sheep) OR (ovine)) NOT (review)
- ((myasthenia gravis[MeSH Terms]) OR (myasthenia gravis)) AND ((nivolumab) OR (ONO-4538) OR (BMS-936558) OR (MDX1106)) AND ((pig) OR (suine) OR (horse) OR (equine) OR (cow) OR (cattle) OR (bovine) OR (sheep) OR (ovine)) NOT (review)
- ((myasthenia gravis[MeSH Terms]) OR (myasthenia gravis)) AND ((pembrolizumab) OR (MK-3475) OR (lambrolizumab)) AND ((pig) OR (suine) OR (horse) OR (equine) OR (cow) OR (cattle) OR (bovine) OR (sheep) OR (ovine)) NOT (review)
- ((myasthenia gravis[MeSH Terms]) OR (myasthenia gravis)) AND ((atezolizumab) OR (MPDL3280A) OR (RG7446)) AND ((pig) OR (suine) OR (horse) OR (equine) OR (cow) OR (cattle) OR (bovine) OR (sheep) OR (ovine)) NOT (review)
- ((myasthenia gravis[MeSH Terms]) OR (myasthenia gravis)) AND ((durvalumab) OR (MEDI4736) OR (MEDI-4736)) AND ((pig) OR (suine) OR (horse) OR (equine) OR (cow) OR (cattle) OR (bovine) OR (sheep) OR (ovine)) NOT (review)
- ((myasthenia gravis[MeSH Terms]) OR (myasthenia gravis)) AND ((avelumab) OR (MSB0010718C)) AND ((pig) OR (suine) OR (horse) OR (equine) OR (cow) OR (cattle) OR (bovine) OR (sheep) OR (ovine)) NOT (review)
- ((myasthenia gravis[MeSH Terms]) OR (myasthenia gravis)) AND ((tremelimumab) OR (ticilimumab)) AND ((pig) OR (suine) OR (horse) OR (equine) OR (cow) OR (cattle) OR (bovine) OR (sheep) OR (ovine)) NOT (review)
- ((myasthenia gravis[MeSH Terms]) OR (myasthenia gravis)) AND (pidilizumab) AND ((pig) OR (suine) OR (horse) OR (equine) OR (cow) OR (cattle) OR (bovine) OR (sheep) OR (ovine)) NOT (review)
- ((myasthenia gravis[MeSH Terms]) OR (myasthenia gravis)) AND ((cemiplimab) OR (REGN-2810) OR (REGN2810) OR (cemiplimab-rwlc)) AND ((pig) OR (suine) OR (horse) OR (equine) OR (cow) OR (cattle) OR (bovine) OR (sheep) OR (ovine)) NOT (review)

Guinea pig/baboon/macaque/chimpanzee/chicken/fish:

- ((myasthenia gravis[MeSH Terms]) OR (myasthenia gravis)) AND (immune checkpoint) AND ((guinea pig) OR (baboon) OR (macaque) OR (chimpanzee) OR (chicken) OR (fish)) NOT (review)
- ((myasthenia gravis[MeSH Terms]) OR (myasthenia gravis)) AND ((anti-PD-1) OR (anti-PD-L1) OR (anti-CTLA-4)) AND ((guinea pig) OR (baboon) OR (macaque) OR (chimpanzee) OR (chicken) OR (fish)) NOT (review)
- ((myasthenia gravis[MeSH Terms]) OR (myasthenia gravis)) AND ((ipilimumab) OR (BMS-734016) OR (MDX-010) OR (MDX-101)) AND ((guinea pig) OR (baboon) OR (macaque) OR (chimpanzee) OR (chicken) OR (fish)) NOT (review)
- ((myasthenia gravis[MeSH Terms]) OR (myasthenia gravis)) AND ((nivolumab) OR (ONO-4538) OR (BMS-936558) OR (MDX1106)) AND ((guinea pig) OR (baboon) OR (macaque) OR (chimpanzee) OR (chicken) OR (fish)) NOT (review)
- ((myasthenia gravis[MeSH Terms]) OR (myasthenia gravis)) AND ((pembrolizumab) OR (MK-3475) OR (lambrolizumab)) AND ((guinea pig) OR (baboon) OR (macaque) OR (chimpanzee) OR (chicken) OR (fish)) NOT (review)
- ((myasthenia gravis[MeSH Terms]) OR (myasthenia gravis)) AND ((atezolizumab) OR (MPDL3280A) OR (RG7446)) AND ((guinea pig) OR (baboon) OR (macaque) OR (chimpanzee) OR (chicken) OR (fish)) NOT (review)
- ((myasthenia gravis[MeSH Terms]) OR (myasthenia gravis)) AND ((durvalumab) OR (MEDI4736) OR (MEDI-4736)) AND ((guinea pig) OR (baboon) OR (macaque) OR (chimpanzee) OR (chicken) OR (fish)) NOT (review)
- ((myasthenia gravis[MeSH Terms]) OR (myasthenia gravis)) AND ((avelumab) OR (MSB0010718C)) AND ((guinea pig) OR (baboon) OR (macaque) OR (chimpanzee) OR (chicken) OR (fish)) NOT (review)
- ((myasthenia gravis[MeSH Terms]) OR (myasthenia gravis)) AND ((tremelimumab) OR (ticilimumab)) AND ((guinea pig) OR (baboon) OR (macaque) OR (chimpanzee) OR (chicken) OR (fish)) NOT (review)
- ((myasthenia gravis[MeSH Terms]) OR (myasthenia gravis)) AND (pidilizumab) AND ((guinea pig) OR (baboon) OR (macaque) OR (chimpanzee) OR (chicken) OR (fish)) NOT (review)
- ((myasthenia gravis[MeSH Terms]) OR (myasthenia gravis)) AND ((cemiplimab) OR (REGN-2810) OR (REGN2810) OR (cemiplimab-rwlc)) AND ((guinea pig) OR (baboon) OR (macaque) OR (chimpanzee) OR (chicken) OR (fish)) NOT (review)

Paraneoplastic MG/LEMS:

- ((myasthenia gravis[MeSH Terms]) OR (myasthenia gravis) OR (lambert-eaton myasthenic syndrome) OR (lambert eaton myasthenic syndrome) OR (lambert-eaton syndrome) OR (lambert eaton syndrome)) AND (paraneoplastic)
- ((myasthenia gravis[MeSH Terms]) OR (myasthenia gravis)) AND ((thymus) OR (thym*))
- ((lambert-eaton myasthenic syndrome) OR (lambert eaton myasthenic syndrome) OR (lambert-eaton syndrome) OR (lambert eaton syndrome)) AND ((SCLC) OR (small cell lung cancer))

**Immune checkpoint inhibitors included:**

- Anti-PD-1, anti-PD-L1, and anti-CTLA-4
- Ipilimumab
- Nivolumab
- Pembrolizumab
- Toripalimab
- Atezolizumab
- Durvalumab
- Avelumab
- Tremelimumab
- Pidilizumab
- Cemiplimab
- Toripalimab

**Species included:**

- Humans
- Mice/rats/rodents
- Dogs
- Cats
- Pigs
- Horses
- Sheep
- Cattle
- Guinea pigs
- Baboons/macaques/chimpanzees
- Chicken
- Fish

**Integrated Metric of Evidence Calculation and Score Deviation:**

For each immune checkpoint inhibitor identified in a paper, an integrated metric of evidence (IME) will be computed as follows: IME = 2D + Q + 2L + I + N; where study design (D) and quality (Q), likelihood of a causal link between immune checkpoint inhibitors and MG (L), confidence of MG diagnosis (I), and the number of patients treated with given immune checkpoint inhibitor (N) will be assessed. A maximum normalized score of 1 will be assigned for each value, yielding a total maximum score of 7.

*D score:* A score D, from 1 (lowest) to 7 (highest) will be given based on whether the study addresses one of the questions “Does the immune checkpoint inhibitor induce (or is associated with) MG as a part of the hypothesis or specific aims?” or “Is the question that an immune checkpoint inhibitor induces (or is associated with) MG answered by study design?” If the answer is no, the study will be described as “Descriptive Association Only” and will be assigned the lowest score of 1. If the answer is yes, a D score will be assigned from 2 to 7. A “Retrospective case series or case report” will be assigned 2 points; a “Cross-sectional study” will be given 3 points; a “Retrospective cohort or case-control study” will be assigned 4 points; both “Prospective cohort study” and “Prospective case-control study” will be given 5 points; an “Unblinded randomized or non-randomized control trial or experiment” will be assigned a score of 6; a “Blinded randomized control trial or experiment” will be assigned the highest score of 7. A normalized D score will be derived by dividing the assigned score by 7.

| **Type of study** | **Description** |
| --- | --- |
| Blinded randomized control trial or experiment (prospective) | A blinded randomized control trial (RCT) is one in which cases are allocated at random to receive one of several clinical interventions. One of these interventions is the standard of comparison or control. The control may be a standard practice, a placebo, or no intervention at all. RCTs seek to measure and compare the outcomes after the participants receive the interventions. Since the outcomes are measured, RCTs are quantitative studies. In sum, RCTs are quantitative, comparative, controlled experiments (i.e. prospective in nature) in which investigators study two or more interventions in a series of individuals who receive them in random order. |
| Unblinded, or unrandomized, control trial or experiment (prospective) | As above, but not blinded - i.e. the investigators know which is the experimental and which is the control group. |
| Prospective cohort or case-control study | A cohort study is a quasi-experiment in the form of a longitudinal study (generally a type of observational study). In a cohort study there is a passive follow-up of a group of animals and documentation of relevant characteristics or events related to this group; note that inclusion of information on survival defines a cohort study for our purposes. A cohort study involves an analysis of risk factors and follows a group of animals that do not have the disease, using correlations to determine the absolute risk of developing disease. Cohort studies are largely about the life histories of segments of populations, and the individual animals within these segments. A cohort is a group of animals sharing a common characteristic or experience within a defined period. The comparison group may be the general population from which the cohort is drawn, or it may be another cohort of animals thought to have experienced little or no exposure to the factor under investigation, but otherwise similar. Alternatively, subgroups within the cohort may be compared with each other. Cohort studies may be conducted prospectively, or retrospectively from archived records. |
| Retrospective cohort or case-control study | See above: the study is a cohort or case-control study, but based upon archived records |
| Cross-sectional study | A cross-sectional study is a type of observational study that involves the analysis of data collected from a population, or a representative subset, at ONE specific point in time — that is, cross-sectional data. Cross-sectional studies differ from case-control studies in that they aim to provide data on the entire population under study, whereas case-control studies typically include only individuals with a specific characteristic, comparing them with a sample, often a tiny minority, of the rest of the population. Cross-sectional studies are descriptive studies (neither longitudinal nor experimental). Unlike case-control studies, they can be used to describe not only the odds ratio, but also absolute risks and relative risks from prevalences. They may be used to describe some feature of the population, such as prevalence of an illness, or they may support inferences of cause and effect. At one point in time the subjects are assessed to determine whether they were exposed to the relevant factor and whether they have the outcome of interest. Some of the subjects will not have been exposed nor have the outcome of interest. This clearly distinguishes this type of study from the other observational studies (cohort and case-control), in which reference to either exposure and/or outcome is made. |
| Retrospective case series or case report | A case series tracks subjects with a known exposure, such as animals that have received a similar treatment, or examines their medical records for exposure and outcome. Case series may be consecutive or non-consecutive, depending on whether all cases presenting to the reporting authors over a time period were included, or only a selection. Case series have a descriptive study design: unlike studies that employ an analytical design (e.g. case-control or cohort studies, or RCTs), case series do not involve hypothesis testing to look for evidence of cause and effect, but may be used to speculate on such associations. Case series are especially vulnerable to selection bias: only the presence of a comparator group, which is not a feature of case-series studies, will allow a valid estimate of true treatment effect. Case reports provide information on only a single case. |

*Q score:* Q score will be assigned as 0 for studies described as “Descriptive Association Only” since it does not address the research question regarding a causal relationship between drug or vaccine and MG. Quality of the articles will be assessed based on the following questions:

- Q1: Is/are the study hypothesis/hypotheses that an immune checkpoint inhibitor induces/is associated with MG or LEMS clearly stated, OR is the question that an immune checkpoint inhibitor induces/is associated with MG or LEMS clearly answered by study design?
- Q2: Is/are the specific aim(s)/objective(s) of the study clearly stated AND does at least one aim/objective include a means to identify whether an immune checkpoint inhibitor induces/is associated with MG or LEMS?
- Q3: Does the study involve multiple hospitals, clinics, and/or research institutions?
- Q4: Does the study include clear inclusion/exclusion criteria?
- Q5: Are there data on the cases screened and excluded from the study?
- Q6: Are the search terms/keywords described?
- Q7: Are the results clearly and objectively presented?
- Q8: Are appropriate statistical tests used? (Is data analysis included and are the appropriate statistical tests implemented for the study design that do not bias the results?)
- Q9: Is the measure of variability reported?
- Q10: Is the conclusion supported by the reported results?
- Q11: Is there a clear conflict of interest/disclosure statement?

Answers to all questions will be “No/Absent/Unclear/NA” (assigned 0 points), “Partially reported/suggested” (assigned 1 point), or “Yes” (assigned 2 points).

A weighted sum of these 11 questions will be computed based on the following equation: 2(Q1) + 2(Q2) + Q3 + Q4 + Q5 + Q6 + 3(Q7) + 3(Q8) + 2(Q9) + 3(Q10) + Q11. A normalized Q score will be calculated by dividing the assigned score by 40.

*L score:* L scores will address the causal link between administration of immune checkpoint inhibitor and MG. “No/Absent/Unclear/NA” causal link will be assigned a score of 1, a “Partially reported/suggested temporal association” causal link will be assigned a score of 2, a “Partially reported/suggested immunopathogenic mechanism” causal link will be assigned a score of 3, and a “Yes/Confirmed” causal link will be assigned a score of 4. A normalized L score will be calculated by dividing the assigned score by 4.

| **Answer** | **Description** |
| --- | --- |
| Yes/Confirmed | Prospective experimental evidence of causality, which is likely to include mechanistic dissection of immunopathogenesis |
| Partially reported/suggested immunopathogenic mechanism | Ending of administration of drug leads to clinical improvement or remission of MG OR demonstration of possible immunopathogenic mechanism(s) without prospective experimental interrogation |
| Partially reported/suggested temporal association | Onset of MG symptoms within 12 weeks of administration of ICI therapy |
| No/absent/unclear/NA | All other observations |

*I score:* I scores will address the confidence of MG diagnosis. “Supportive” will be given 1 point, “Diagnostic” (serological) will be given 2 points, and “Mechanistic study” will be given 3 points. A normalized I score will be calculated by dividing the assigned score by 3.

| **Answer** | **Description** | **Search Term** |
| --- | --- | --- |
| Mechanistic Study | Induction of experimental autoimmune myasthenia gravis (EAMG) | (“Experimental autoimmune myasthenia gravis” OR “EAMG”) AND ((“Mouse” OR “Mice”) OR (“Rat”) |
| Diagnostic | Positive serological identification of anti-AChR antibodies or anti-MuSK antibodies or anti-VGCC autoantibodies (titer or ELISA) | “AChR antibod*” OR “Acetylcholine receptor antibod*” OR “Muscle specific tyrosine kinase receptor antibod*” OR “MuSK antibod*” OR “Voltage gated calcium channel antibod*” OR “VGCC antibod*” |
| Supportive | Electrophysiological study findings or positive serological identification of anti-striational antibodies | “Electrophysiology” OR “Electromyogram” OR “repetitive nerve stimulation” OR “anti-striational” |

*N score:* N score will be given based on the number of patients treated with an immune checkpoint inhibitor who developed myasthenia gravis, where 1 point will be assigned for 1 patient, 2 points for 2-5 patients, 3 points for 6-10 patients, 4 points for 11-20 patients, 5 points for 21-50 patients, and 6 points for ≥51 patients. A normalized N score will be calculated by dividing the assigned score by 6.
